# Natural phytochemicals prevent side effects in *BRCA*-mutated ovarian cancer and PARP inhibitor treatment

**DOI:** 10.3389/fphar.2022.1078303

**Published:** 2022-12-07

**Authors:** Chuanlin Wang, Pengning Gao, Jiali Xu, Shanling Liu, Wenda Tian, Jiayu Liu, Lan Zhou

**Affiliations:** ^1^ Department of Clinical Nutrition, Yunnan Cancer Hospital, The Third Affiliated Hospital of Kunming Medical University, Kunming, Yunnan, China; ^2^ Yunnan Cancer Center, Kunming, Yunnan, China; ^3^ Department of Gynecology, Yunnan Cancer Hospital, The Third Affiliated Hospital of Kunming Medical University, Kunming, Yunnan, China; ^4^ Key Laboratory of Environmental Toxicology of Anhui Higher Education Institutes, Department of Toxicology, School of Public Health, Anhui Medical University, Hefei, Anhui, China

**Keywords:** phytochemicals, ovarian cancer, PARP, *BRCA*, PARP inhibitors

## Abstract

Ovarian cancer is among the most common malignant tumors in gynecology and is characterized by insidious onset, poor differentiation, high malignancy, and a high recurrence rate. Numerous studies have shown that poly ADP-ribose polymerase (PARP) inhibitors can improve progression-free survival (PFS) in patients with *BRCA*-mutated ovarian cancer. With the widespread use of *BRCA* mutation and PARP inhibitor (PARPi) combination therapy, the side effects associated with *BRCA* mutation and PARPi have garnered attention worldwide. Mutations in the *BRCA* gene increase KEAP1-NRF2 ubiquitination and reduce Nrf2 content and cellular antioxidant capacity, which subsequently produces side effects such as cardiovascular endothelial damage and atherosclerosis. PARPi has hematologic toxicity, producing thrombocytopenia, fatigue, nausea, and vomiting. These side effects not only reduce patients’ quality of life, but also affect their survival. Studies have shown that natural phytochemicals, a class of compounds with antitumor potential, can effectively prevent and treat the side effects of chemotherapy. Herein, we reviewed the role of natural phytochemicals in disease prevention and treatment in recent years, including sulforaphane, lycopene, catechin, and curcumin, and found that these phytochemicals have significant alleviating effects on atherosclerosis, nausea, and vomiting. Moreover, these mechanisms of action significantly correlated with the side-effect-producing mechanisms of *BRCA* mutations and PARPi. In conclusion, natural phytochemicals may be effective in alleviating the side effects of *BRCA* mutant ovarian cancer cells and PARP inhibitors.

## 1 Introduction

Ovarian cancer is among most common malignancies in gynecology (Bookman et al., 2009). Two hundred thousand women worldwide are diagnosed with ovarian cancer each year, 70% of whom are intermediate to advanced cases, with a mortality rate of 62.5%. High-grade plasmacytoma is a common type of ovarian cancer that arises from ovarian epithelial cells. It is poorly differentiated, highly malignant, and has a high recurrence rate (Colombo et al., 2019). According to treatment guidelines, ovarian cancer is treated chiefly with platinum drugs in combination with paclitaxel or with the anti-angiogenic drug bevacizumab alone (Perren et al., 2011). Platinum drugs are key to treating platinum-sensitive recurrent ovarian cancer; however, as the number of recurrences increases, this type of ovarian cancer becomes resistant to platinum drugs (Foley et al., 2013). The median progression-free survival (PFS) for bevacizumab was 19.0 months, slightly higher than the median PFS in the standard treatment group (17.3 months), as noted by the European Society of Medical Oncology and the International Society for Gynecologic Cancer meetings (Perren et al., 2011). Although rational treatment significantly prolongs patient survival, 7080% of patients experience relapse or further disease progression after first-line treatment (Lorusso et al., 2020). PARPi, an inhibitor of polyadenosine diphosphate ribose polymerase, extends PFS to approximately 56 months in patients with platinum-resistant, BRCA-deficient, or refractory ovarian cancer by affecting the self-replication of ovarian cancer cells, providing a new approach for the maintenance treatment of ovarian cancer patients (Mirza et al., 2019; Vanacker et al., 2021).

Numerous studies have demonstrated that phytochemicals extracted from foods have antitumor potential. Audesh et al. found that some phytochemicals extracted from fruits have significant inhibitory effects on human ovarian teratoma cells (PA-1) at their respective IC50 concentrations (Li et al., 2021). Phytochemicals have been extensively studied to inhibit the development of ovarian cancer, and interfere with cancer cells along with antioxidant and anti-inflammatory effects (Pundir et al., 2021). Islam et al. demonstrated that the antioxidant and anti-inflammatory effects of phytochemicals were effective in preventing some side effects of chemotherapy (Islam et al., 2021). Chemotherapy plays a very important role in ovarian cancer treatment, but its side effects also seriously affect patients’ quality of life, and symptomatic supportive treatment to alleviate these side effects will further increase the burden on the patient’s body. In contrast to drugs, various types of phytochemicals, such as phenols, terpenoids, and sulfur-containing compounds, are distributed in numerous fruits and vegetables consumed daily (Roy and Datta, 2019). The use of phytochemicals as an alternative to drugs would reduce the patient’s fear and organismal burden of oncologic chemotherapy and improve patient compliance.

This study reviewed the mitigating effects of phytochemicals on PARPi side effects and the prevention of pathological changes caused by *BRCA* mutations. We further clarified the mechanisms by which phytochemicals alleviate the side effects of synergistic lethal treatment regimens.

## 2 PARP, PARPi, and ovarian cancer

### 2.1 PARP and ovarian cancer

Poly (adenosine diphosphate ribose) polymerase (PARP) is a cleavage substrate for the core members of apoptosis, caspases. PARP is also involved in damage repair after DNA breaks (Wei and Yu, 2016). PARP1 plays more than 90% of the total role of the PARP family, and PARP1 is active in base excision repair (BER) (Durkacz et al., 1980), DNA single-strand breaks (SSB) (Haince et al., 2008), DNA double-strand breaks (DSBs), and replication fork damage (Haince et al., 2008). After DNA damage, PARP1 recognizes the damage through the zinc finger structural domain and orientates to the nick for ADP ribosylation based on nicotinamide adenine dinucleotide (NAD+), forming PARP-1-ADP ribose branched chain, which reduces the binding of PARP1 to DNA and dissociates from DNA to participate in DNA repair (Bürkle, 2001; Lord and Ashworth, 2017), PARP2 is similar to PARP1 in function, but it acts on different substrates (Kutuzov et al., 2020), and PARP2 is crucial in the repair of SSBs. It is by damaging DNA and thus affecting mitosis that cisplatin treats ovarian cancer. Thus, PARP plays a key role in apoptosis and repair of platinum-induced DNA damage in ovarian cancer cells (Hoeijmakers, 2001; Damia and Broggini, 2019).

### 2.1 PARPi and ovarian cancer

PARPi competes with NAD+ for the PARP active site, thereby inhibiting the formation of poly (ADP-ribose) polymers; when single-strand damage occurs in DNA molecules, the repair is mainly accomplished by PARP and DNA ligase IIIa (Murai et al., 2012; Murai et al., 2014). PARPi can bind specifically to the NAD+ binding site of PARP1 (and/or PARP2), resulting in a significant reduction in DNA-PARP dissociation, maintaining PARP binding to DNA, thus perpetuating the DNA-PARP complex and inhibiting subsequent repair. This process is known as “trapping” of the DNA-PARP complex (Murai et al., 2012). The persistence of the complex on a single strand of DNA allows for the accumulation of large amounts of single-stranded broken DNA and thus DSBs, causing cell death. To resolve these barriers and restore the cell cycle, functional homologous recombination (HR) must be utilized (Bunting et al., 2010).

## 3 *BRCA* gene mutation, PARPi, and ovarian cancer

### 3.1 *BRCA* gene mutations and ovarian cancer

Breast cancer susceptibility gene (*BRCA*) participates in DNA repair and is present in the human body as a tumor suppressor gene (Prakash et al., 2015). Carriers of *BRCA1* and *BRCA2* germline mutations have a 54% and 23% risk, respectively, of developing ovarian cancer (Ramus and Gayther, 2009; Milne and Antoniou, 2011). First, *BRCA* proteins act through the HR process to protect humans (Bryant et al., 2005). HR ensures that the cellular repair of DSBs in the S-phase is precise and error-free (Farmer et al., 2005). The function of *BRCA1* in HR is to cleave DSB 5′–3′, leaving an overhanging 3′. HR is an essential method of DNA double-strand break repair. The HR repair pathway is purportedly blocked by *BRCA* (*BRCA1/2*) mutations. In this case, the DSB repair mechanism is no longer stable and the DNA damage repair function of the cell is greatly reduced. Therefore, cancer cells damaged by platinum cannot be repaired (Bryant et al., 2005; D’Andrea, 2018; Li et al., 2020). This suggests that BRCA plays a role in the repair of DSBs (Gudmundsdottir and Ashworth, 2006). The application of platinum-based drugs after *BRCA* mutations can inhibit DNA replication in ovarian cancer cells (Birkbak et al., 2012).

### 3.2 Synergistic lethal effects of *BRCA* mutations and PARPi in ovarian cancer

**FIGURE 1:**
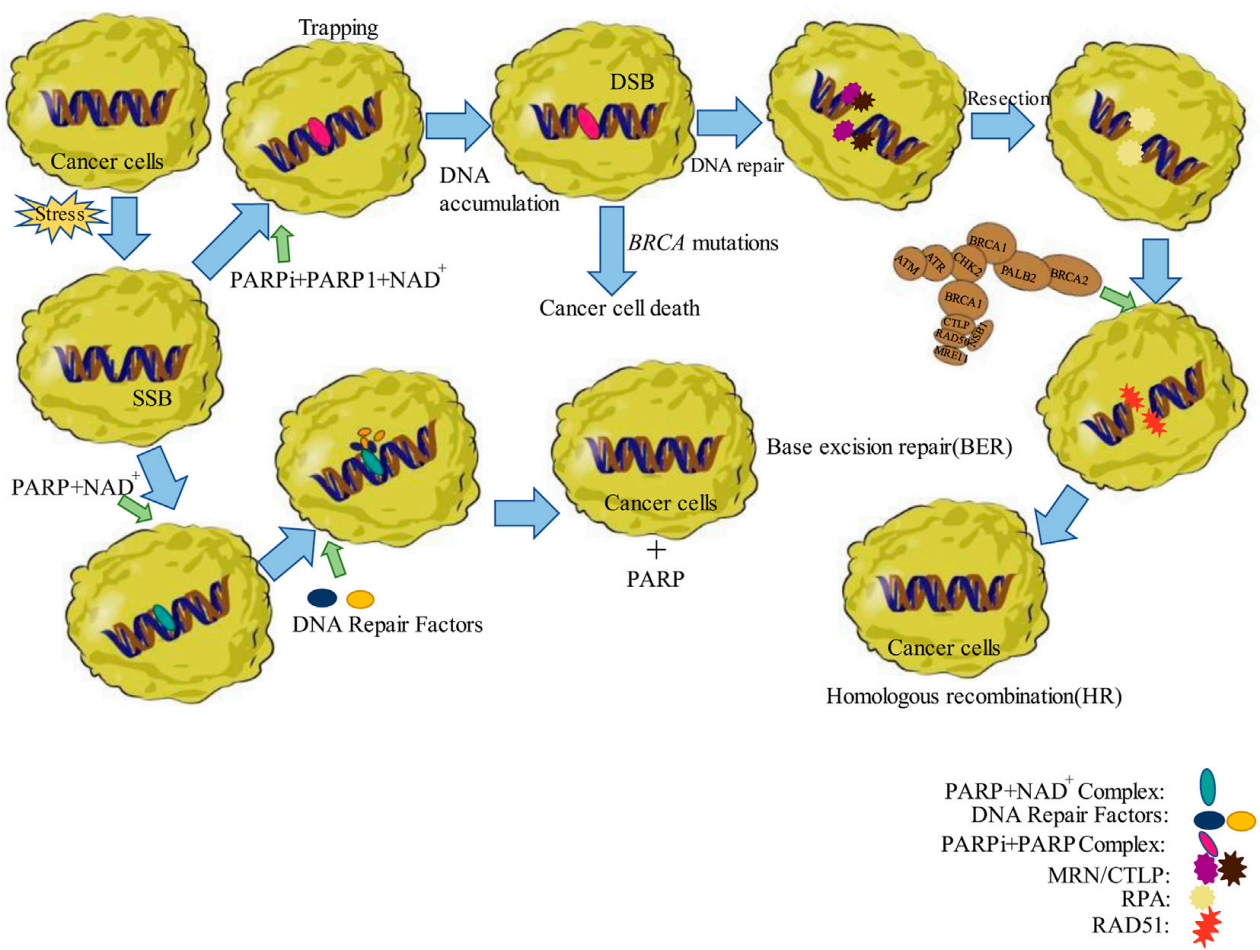
Schematic representation of the functions of PARP and *BRCA* in DNA repair. The left side shows base excision repair. Right side shows the “trapping” of the DNA-PARP complex and DNA homologous recombination repair. Abbreviations: DSB, double-strand break; MRN/CTLP, DNA damage sensor; NAD+, nicotinamide adenine dinucleotide; RAD51, restriction-associated site DNA51; RPA, replication proteinase A; SSB, single-strand break.

If PARPi is used in the presence of *BRCA* mutations in ovarian or breast cancer cells, then, it will further inhibit DNA break repair due to HR defects, and the cells will be unable to repair DSBs leading to cell death, a synergistic lethal phenomenon (Rottenberg et al., 2008; Srinivasan et al., 2017). This phenomenon destabilizes the tumor genome, which can counteract the tumor cell proliferation and effectively increase the patients’ survival time. Therefore, PARPi induces cell death in HR-deficient cells as a primary approach for the treatment of ovarian cancer (Noordermeer and van Attikum, 2019; Curtin and Szabo, 2020) as shown in Figure 1.

## 4 Mitigation of adverse drug reactions to PARPi by phytochemicals

### 4.1 Side effects of PARPi

The use of PARPi in patients with *BRCA*-deficient ovarian cancer has had notable success, but the use of PARPi induces discomfort in ovarian cancer patients. For example, the hematologic toxicity produced by niraparib (ZEJULA), a highly absorbed, highly permeable drug, should not be underestimated. Berek observed in 553 patients who added niraparib, that about 33% developed thrombocytopenia and 13% developed anemia (Berek et al., 2018). Meanwhile, data published by LaFargue et al. (2019) showed that the probability of fatigue in the first month after niraparib was approximately 32.4%, vomiting was about 19.6%, and nausea was up to 61.9%. Most of the fatigue was due to ischemia and decreased platelet count. Olaparib (Lynparza), a low permeability, low absorption drug, is highly susceptible to hypertension, with a 48% chance of nausea and vomiting. The side effects of PARPi seriously affect patients’ quality of life (Munroe and Kolesar, 2016; Paik, 2021).

### 4.2 Mitigation of PARPi side effects by phytochemicals

Phytochemicals have strong antioxidant properties and are commonly used for skin care. However, numerous studies have shown that saffron, cyclic adenosine phosphate, and curcumin from ginger can reduce the incidence of some chemotherapy side effects, such as nausea, vomiting, and anemia, at the sites shown in Table 1.

**TABLE 1:** The side effect loci of phytochemistry in the prevention of *BRCA* mutations and PARPi.

Category	Name	Mode of action	Site of action
Sulfur-containing compounds			
	Sulforaphane	Interferents	Keap1, Nrf2
	Allicin	Supplements	GSH, GPX
Terpenoids			
	Lycopene	Regulatory proteins	P62, Keap1, Nrf2
	Lutein	Regulatory proteins	ERK, Nrf2
Polyphenols			
	Catechins	Agonists	CAT, GSH
	Proanthocyanidins	Ca^+^ regulation	NO, SOD2, GPX, NOX4
	Quercetin	o-Diphenol hydroxyl	-OH, O2-
Polyphenols			
	Anthocyanin	For electronics	Free radicals
	Soy isoflavones	For hydrogen atoms	Free radicals
	Curcumin	Regulatory proteins	miR-125b, HAT
	Ginger	Interferents	5-HT
	Cyclic adenosine monophosphate	Agonist	Erythropoietin
	Crocin	Interferon	Platelets

#### 4.2.1 Crocin

Crocin, a carotenoid present in the stigma of saffron, improves collagen-induced platelet aggregation and adhesion and A23187-mediated endogenous production of ROS and H_2_O_2_ in platelet mitochondria (Thushara et al., 2013; Yaribeygi et al., 2018). Pourmasoumi et al. (2019) reported significant decreases in diastolic blood pressure, body weight, and other factors associated with cardiovascular disease (CVD) in 622 individuals taking Crocin. Javandoost et al. (2017) found that adding Crocin was associated with a significant increase in high-density lipoprotein (HDL) levels during an 8-week Crocin intervention. The addition of Crocin to PARPi not only reduces oxidative stress but also prevents the reduction of platelets and increases blood pressure. Crocin also reduces HDL production, which can reduce the prevalence of CVD in several ways.

#### 4.2.2 Adenosine cyclic phosphate

The high content of cyclic adenosine phosphate in jujube can dilate blood vessels, provide nutrients to the heart muscle and increase its contractility, induce the expression of erythropoietin, and stimulate hematopoiesis in the body (Chen and Tsim, 2020). The increase in hematopoietic parameters after cancer treatment in mice with jujube further suggests that jujube can ameliorate anemia in cancer patients (Periasamy et al., 2020). Improvement in anemia results in patients feeling less fatigued. The cyclic adenosine phosphate in dates may reduce the discomfort experienced by patients after niraparib administration.

#### 4.2.3 Ginger active substance

The use of ginger as an antiemetic is well-known in China and is used in traditional Chinese medicine, where ginger is rich in curcumin, curcumin, gingerols, and curcuminoids (Ahmed et al., 2021). These active substances influence gastrointestinal motility and promote gastric emptying, while they affect the central nervous system by mediating the 5-hydroxytryptamine-3 of 5-hydroxytryptamine (5-HT), reducing nausea and vomiting (Nocerino et al., 2021). Marx et al. (2017) conducted a double-blind randomized intervention with ginger in 51 patients, identifying less fatigue in the intervention group (*p* = 0.006) from the three chemotherapy cycles, especially in the third cycle. Subsequently, Crichton et al. (2019) found through a meta-analysis that ginger supplementation not only had a significant effect in suppressing nausea and vomiting but also reduced the likelihood of fatigue by approximately 80%. Therefore, the administration of ginger in the treatment of ovarian cancer with PARPi can reduce PARPi side effects in patients.

**FIGURE 2:**
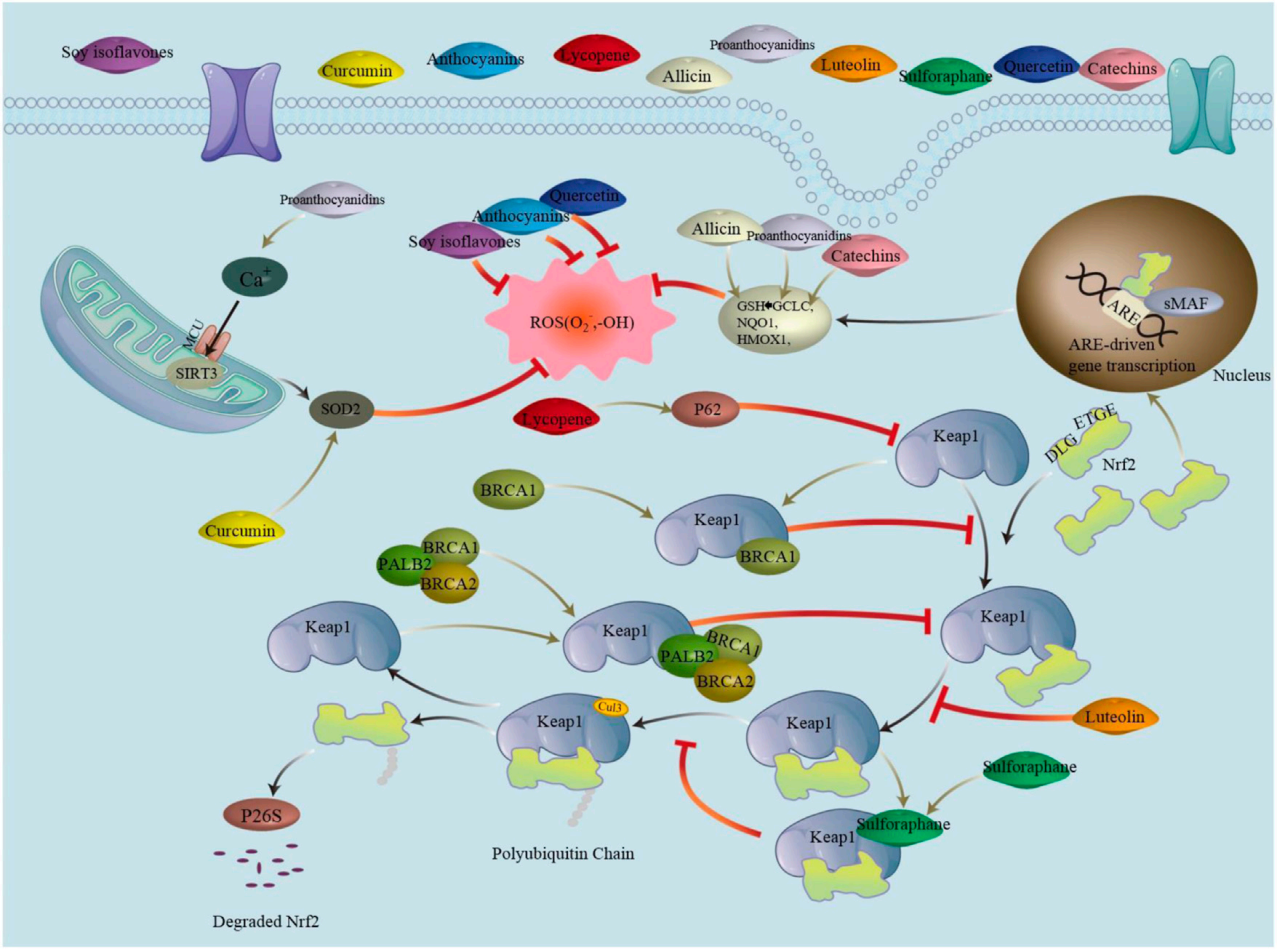
Nrf2/Keap1 is a signaling pathway in which Nrf2 binds to Keap1 *via* ETGE and DLG, ubiquitinating it, which is then degraded by the proteasome. Phytochemicals can promote nuclear translocation of Nrf2 by mediating the Keap1-Nrf2 complex. Binding ARE after forming a heterodimer with sMAF activates transcriptional production of downstream antioxidant enzymes. Phytochemicals can also affect ROS production by acting directly on ROS. Abbreviations: ARE, antioxidant response element; *BRCA* (1/2), breast cancer 1/2; CUL3, cullin3; DLG/ETGE, nrf2 structural domain; Keap1, recombinant kelch like ech associated protein1; MCU, mitochondrial calcium uniporter; Nrf2, nuclear factor erythroid 2-related factor 2; PALB2, partner and localizer of brca2; ROS, reactive oxygen species; SIRT3, silence regulatory protein3; sMAF, specific macrophage arming factor; SOD2, superoxide dismutase.

Saffron, cyclic adenosine phosphate, and curcumin have a significant inhibitory effect on the side effects caused by niraparib and olaparib, as shown in Figure 2. In the future, a rational combination could reduce the pain associated with treatment of ovarian cancer patients and increase their quality of life.

## 5 Phytochemicals attenuate adverse effects in *BRCA* mutations synergistically lethal with PARPi

### 5.1 *BRCA* mutations cause cardiovascular disease

Mutations or deletions of *BRCA* in normal individuals significantly increase the risk of developing cancers, such as ovarian cancer (Sekine et al., 2021). However, diseases beyond ovarian or breast cancer are associated with *BRCA*, and analysis excluding causes of cancer death found that survival was also significantly lower in individuals with mutations or deletions in *BRCA* (Mai et al., 2009).

Many survival studies on *BRCA* gene deletions have sufficiently demonstrated that cardiovascular-related diseases are another critical cause of death in individuals with *BRCA* mutations or deletions (Arts-de Jong et al., 2014; Lammert et al., 2022). Sajjad et al. (2017) studied 401 cancer-free female *BRCA1/2* mutation carriers and found that *BRCA* mutation carriers were at increased risk of cardiovascular disease compared to the general population. Zhou et al. noted that *BRCA* gene deletion causes cardiac diseases including ischemic heart disease, atherosclerosis, and other myocardial diseases (Arts-de Jong et al., 2014). Atherosclerosis is a major cause of aortic disease, peripheral vascular-related diseases, coronary heart disease, and cerebral infarction (Alexander et al., 2021; Shea et al., 2021). Therefore, addressing atherosclerosis is key to preventing cardiovascular diseases caused by *BRCA* defects.

Atherosclerosis has been extensively studied, and through the analysis of causative factor ranking, endothelial dysfunction has been established as the factor of atherosclerosis (Gimbrone and García-Cardeña, 2016), and apoptosis of endothelial cells plays a crucial role in the occurrence of endothelial dysfunction (Xu et al., 2021), thus, can be suggested that endothelial cell injury plays a driving role in atherosclerosis (Zheng et al., 2017). Therefore, inhibiting endothelial cell apoptosis in atherosclerosis can help prevent atherosclerosis development (Gimbrone and García-Cardeña, 2016).

Low-density lipoprotein (LDL) represents the beginning of the atherosclerotic response when it enters the subendothelial space from the endothelium by cellular action and is deposited in the subintima of the vessel where it is oxidized by ROS (Porter et al., 2013). Oxidation of LDL by ROS results in the formation of oxidized low-density lipoprotein (Ox-LDL), which is accompanied by endothelial destruction, binding of Ox-LDL to the scavenger receptors of macrophages, and intracellular accumulation of Ox-LDL after phagocytosis by vascular smooth muscle cells, resulting in the formation of foam cells (Porter et al., 2013). ROS cause endothelial cell apoptosis and atherosclerosis. Therefore, ROS can be used as both a marker of early atherosclerosis and as an entry point to control atherosclerosis (Panieri and Santoro, 2015). In Korea, Lee et al. (2021) used zearalenone (ZEN) to treat endothelial cells, and the rise of ROS after the activation of cytoplasmic calcium by ZEN further accelerated the apoptosis of endothelial cells, verifying that the difficulty in solving atherosclerosis lies in the treatment of LDL and ROS.

### 5.2 *BRCA* mutations affect atherosclerosis through Nrf2-mediated reactive oxygen species

*BRCA1* regulates ROS as a newly identified Nrf2 (antioxidant transcription factor) binding protein (Vurusaner et al., 2012). In 2006, PALB2 was identified as a protein that interacts with *BRCA2* (Xia et al., 2006). *BRCA1*, *BRCA2,* and *PALB2* are involved in regulating the activity of Keap1 (KELCH-like ECH-associated protein 1)-mediated ubiquitination of Nrf2, thereby regulating the amount of Nrf2, and E3 ubiquitin ligase (cullin3) is a critical enzyme in the ubiquitination reaction, with Keap1 as its recognition subunit (Song et al., 2021). Japanese researchers have found that the ETGE and DLG motifs in the Neh2 structural domain of Nrf2 can bind to the Kelch structural domain of Keap1. ETGE of Nrf2 is bound to the Keap1 dimer using what is known as a hinge, while the Cul3-Rbx1 complex is stably bound to Keap1 using a DLG latching motif (Tong et al., 2006), forming KEAP1-NRF2, which lays the foundation for ubiquitination. Ubiquitinated Nrf2 is then transported to the 26S proteasome to be degraded and destroyed (Zhang et al., 2004). Laboratory analysis of the transfected gene revealed that cells with deletion of the *BRCA1/2* gene are more sensitive to oxidative stress (Fridlich et al., 2015). BRCA1 has an ETGE-like structure, competitively inhibits KEAP1-NRF2 ubiquitination, and increases Nrf2 content by binding to the ETGE-binding site of Keap1 (Zhou et al., 2021). Amino acids 9-44 of *PALB2* determine its linkage to *BRCA1* (Gardini et al., 2014). It was also found that PALB2 is linked to *BRCA2* in the N-terminal domain, and it is worth noting that PALB2 has a highly conserved ETGE-type Keap1 binding motif, which shares the same site of action as Keap1 and Nrf2 (Xia et al., 2007). Thus, *PALB2* can participate in the binding process between Nrf2 and Keap1, compete with Nrf2 for Keap1, inhibit KEAP1-NRF2, and stabilize Nrf2. As Nrf2 mediates the antioxidant response, PALB2 causes Nrf2 to remain in the nucleus to reduce the level of ROS in the cell and the rate of exit from the nucleus (Ma et al., 2012; Gorrini et al., 2013). In the absence of *BRCA1/2* or *PALB2*, KEAP1-NRF2 is not inhibited, ubiquitination of Nrf2 results in high ROS production, and regulating the Keap1 pathway to inhibit endothelial apoptosis and is an essential means of alleviating atherosclerosis from the root (Kobayashi et al., 2004; Singh et al., 2013).

### 5.3 Modulation of *BRCA* mutation-induced cardiovascular lesions by phytochemicals

The prevention of cardiovascular-related diseases through phytochemicals has garnered substantial public interest. Several phytochemicals have been shown to act as cardiovascular disease preventers in cells, animals, and human populations. Examples include sulfur-containing compounds, terpenoids, and polyphenols, the action points of which are listed in Table 1.

#### 5.3.1 Sulfur-containing compounds

##### 5.3.1.1 Sulforaphane

Sulforaphane (SFN), a natural isothiocyanate compound with excellent antioxidant properties, is abundant in cruciferous vegetables and is produced by the breakdown of glucose by endogenous mustard enzymes (Kaiser et al., 2021). Considering the antioxidant properties of SFN, Asif et al. (2022) found that SFN preferentially acts on c151 in Keap1 cysteine residues. In the cytoplasm, Nrf2 binds to Keap1 first due to high ETGE binding, followed by partial binding of DLG, and cullin3 recognizes Keap1 binding immediately, followed by ubiquitination and degradation of Nrf2. If Nrf2 binds to Keap1 and then SFN is added, SFN acts on c151 on Keap1, disrupting the binding of Keap1 to cullin3. Immobilization is prevented and Keap1 cannot continue to participate in the cycle to bind newly generated Nrf2 (Kobayashi et al., 2009). The reduction in Keap1 allows newly generated Nrf2 to enter the nucleus, where it binds to antioxidant response elements (ARE) to activate antioxidant responses, causing a reduction in ROS (Dinkova-Kostova et al., 2017). The regulation of Nrf2 by SFN effectively reduces endothelial cell injury, thus explaining its reduction in atherosclerosis and its role in combating cardiovascular disease (Dana and Alejandro, 2022).

##### 5.3.1.2 Allicin

Glutathione is among the most studied cellular antioxidants. However, orally supplemented glutathione is hydrolyzed and oxidized by intestinal enzymes. Acetylcysteine (NAC) is a precursor of glutathione, and oral supplementation with NAC increases glutathione levels in the body after conversion in the liver (Schmitt et al., 2015). After NAC supplementation, glutathione peroxidase (GPX) activity is enhanced to convert reduced glutathione (GSH) to oxidized glutathione (GSSG), thereby protecting cells from ROS damage (Kwon, 2021). Allicin, also known as diallyl thiosulfate, is a sulfur-containing compound. When allicin was substituted for NAC in intervention studies, researchers also found enhanced GPX activity, which may indicate that allicin, a natural phytochemical, has specific antioxidant effects that counteract ROS production, and thus could be considered for the prevention of atherosclerosis caused by *BRCA* deficiency (Hasan et al., 2006; Catanzaro et al., 2022).

#### 5.3.2 Terpenoids

##### 5.3.2.1 Lycopene

Lycopene (LYC), a terpene fat-soluble natural pigment widely found in tomatoes, watermelon, carrots, and other red fruits and vegetables, can be an effective antioxidant because of its powerful ability to scavenge free radicals LYC induces autophagic degradation of Keap1 by increasing the expression of autophagic protein p62 (Ulasov et al., 2021). When Nrf2 dissociates from Keap1, then nuclear ectopic and binds to ARE in the nucleus to induce the expression of antioxidants downstream of the pathway to avoid oxidative cell death (Baird and Yamamoto, 2020; Wang et al., 2020). Since LYC intervention in rats results in a decrease in LDL and triglycerides and an increase in HDL, it was demonstrated that LYC is an anti-atherogenic phytochemical (Bentzon et al., 2014; Wong, 2014). ROS production was significantly decreased by LYC supplementation, which inhibited endothelial cell injury caused by *BRCA* deletion or mutation (Roy and Datta, 2021). This further demonstrated that LYC is essential for preventing atherosclerosis caused by *BRCA* deficiency.

##### 5.3.2.2 Luteolin

Luteolin, also known as phytoalexin, is among the more common terpene antioxidants in nature that reduces free radical activity, prevents ROS damage to cells, and has a surprising effect on *BRCA*-deficient cancers (Gong et al., 2018). Lutein is an essential nutrient and one of the most common antioxidants found in egg yolks. Furthermore, Mitra et al. (2021) recently noted that dark-colored greens are usually high in luteins, such as kale, spinach, and lettuce. A recent study reconfirmed that the two parts of the carbon chain of lutein are hydrophilic (HO-) and hydrophobic (CH_2_−), respectively (Nakamura and Sugiura, 2022). Moreover, the hydrophilic part of lutein remains on both sides of the cell membrane, whereas the hydrophobic part is in the phospholipid molecule layer, which allows lutein to bind tightly to the cell membrane lipids and increase the stability of the cell membrane (Algan et al., 2022). Conversely, luteolin activates extracellular regulated protein kinase (ERK), allowing Nrf2 phosphorylation and cleavage of the Nfr2/Keap1 complex. This causes nuclear translocation of Nrf2 to bind to the DNA regulatory region of ARE. It induces the expression of antioxidant genes and reduces intracellular ROS levels (Ahn and Kim, 2021). Luteolin can be expressed as an antioxidant that reduces the oxidative response of LDL and inhibits the development of atherosclerosis (Hajizadeh-Sharafabad et al., 2021; Ramanna and Somu, 2021). This suggests that luteolin can inhibit atherosclerosis, thereby preventing the development of CVD.

#### 5.3.3 Polyphenols

Polyphenols significantly impact human health and are known as the “seventh nutrient.” Their role in lowering antioxidant LDL and blood cholesterol has been extensively studied (Abdal Dayem et al., 2016). Vegetables such as spinach, broccoli, and cabbage have high polyphenol contents (Zeb, 2021). Cherries, blueberries, and other dark fruits also have relatively high polyphenol contents. Polyphenols are a natural component of cocoa beans, and the high polyphenol content in black beans contributes to their unique flavor (Yang et al., 2018). Interestingly, Khan et al. (2021) reported that polyphenols not only prevent CVD, but also mediate *BRCA1/2* expression. Polyphenols can be divided into flavonoids and phenolic compounds, the most common of which are catechins, proanthocyanidins, quercetin, soy isoflavones, anthocyanins, and curcumin.

##### 5.3.3.1 Catechins

The antioxidant capacity of catechins is even higher than that of vitamin E. Numerous studies have demonstrated that catechins can increase the activity of antioxidant enzymes (SOD2 and GPX), thus inhibiting the oxidation of LDL to Ox-LDL (Chen et al., 2020; Ahmadi et al., 2022; Dal and Yilmaz, 2022). Japanese researchers observed that LDL oxidation was prolonged in the catechin group by administering 1 g of catechin in capsule form to 19 healthy men in a double-blind crossover trial (Suzuki-Sugihara et al., 2016). The reduction in Ox-LDL levels led to a significant decrease in the probability of atherosclerosis and effectively prevented CVD caused by *BRCA* mutations.

##### 5.3.3.2 Proanthocyanidins

Proanthocyanidins comprise varying amounts of catechins, epicatechin, and gallic acid, which are abundant in grapes and are converted into anthocyanins in plants. Proanthocyanidins play a role in CVD by preventing lipid peroxidation through calcium-dependent NO release, vasorelaxation, and the inhibition of Ox-LDL production (de la Iglesia et al., 2010). Proanthocyanidins reduce intracellular ROS production by increasing the NRF2/Keap1 ratio, increasing SOD2 expression, and inhibiting oxidase expression (NOX4 and iNOS) (Kowalska et al., 2021). In addition, proanthocyanidin supplementation can prevent ROS production from *BRCA* defects (Xian et al., 2019). This reduces the risk of atherosclerosis due to *BRCA* defects.

##### 5.3.3.3 Quercetin

Quercetin is found at high levels in daily life in sea buckthorn, hawthorn, and buckwheat sticks. Its antioxidant capacity is 20 times that of vitamin C and 50 times that of vitamin E. This is due to the good scavenging ability of the o-diphenol hydroxyl group for superoxide anion (O_2_-) and hydroxyl radical (-OH), reducing the production of oxidative stress ROS because the action of the o-diphenol hydroxyl group maintains biofilm integrity (Chu, 2022), and reduces necrosis of vascular endothelial cells. The reduction in ROS leads to the inhibition of LDL oxidation, reducing the risk of atherosclerosis and other cardiovascular diseases (Deng et al., 2020). Concurrently, quercetin inhibits the production of platelet lipoxygenase and cyclooxygenase, which leads to the release of thrombolytic and vascular membrane-protective mediators from the endothelium to counteract thrombosis.

##### 5.3.3.4 Anthocyanins

Anthocyanins are glycosylated anthocyanins that are widely distributed in black, red, and purple plant foods, such as black rice, mulberry, and eggplant, which have powerful antioxidant capacity (Bagchi et al., 2004). Anthocyanins are more substantial than common antioxidants, such as vitamin E, catechins, and quercetin, in scavenging free radicals. They have many phenolic hydroxyl groups, which can directly scavenge many free radicals by oxidizing and releasing electrons to maintain redox balance (Dangles and Fenger, 2018). At the same time, anthocyanins reduce the production of ROS by further activating the activity of SOD2 and GPX to reduce oxidative stress damage (Tian et al., 2019). In addition, it prevents the death of vascular endothelial cells and improves arterial blood-vessel stiffness. In patients with cardiovascular diseases deficient in *BRCA*, supplementation with anthocyanins may improve the risk of related diseases (Speciale et al., 2020).

##### 5.3.3.5 Soy isoflavones

Estrogen secretion increases in ovarian cancer patients (Langdon et al., 2020). When estrogen levels are elevated, the structure of soy isoflavones becomes similar to that of estrogen. Therefore, soy isoflavones prevent estrogen from binding to the receptor, thus acting as estrogen antagonists (Kim, 2021). Moreover, soy isoflavones, similar to quercetin, can contribute to the antioxidant response by providing hydrogen atoms to inhibit the production of reactive oxygen radicals and reduce the level of ROS (Syamala et al., 2021). Su et al. conducted a logistic regression analysis of 500 patients with ovarian cancer and 500 normal subjects (mean age, 59 years) in southern China. They found that moderate intake of soy foods activated cellular autophagy, reduced the risk of ovarian cancer, and increased the sensitivity to carboplatin (Runlin et al., 2022). A Korean study investigated 5509 people at high risk of ovarian cancer and found a relationship between metabolism and soy isoflavone intake, with soy isoflavones being inversely associated with LDL in men and women and negatively associated with the incidence of metabolic syndrome in women. From these data, it can be concluded that soy isoflavone supplementation can inhibit metabolism-induced ROS and LDL production (Woo et al., 2019). Therefore, it is necessary to provide soy isoflavone supplementation to people with *BRCA* mutations, especially to patients with *BRCA* ovarian cancer.

##### 5.3.3.6 Curcumin

Curcumin is a representative phenolic compound and, as a natural compound that can be extracted from the ginger family, deserves our attention as it mediates histone acetyltransferase activity to regulate acetylation of DSB sites, thus reducing the aggregation of critical non-homologous end-joining factors to DSB sites and achieving PARPi sensitization (Ogiwara et al., 2013). Surprisingly, curcumin promotes the increase of ROS in tumor cells, causing tumor cell death (Mortezaee et al., 2019); however, in normal cells, curcumin downregulates the antioxidant response of miR-125b to reduce cell death (Schwertheim et al., 2017). When treating ovarian cancer patients with *BRCA* mutations, adjuvant treatment with curcumin can be considered, not only to increase synergistic lethality, but also to prevent the side effects of PARPi and CVD caused by *BRCA* mutations.

Phytochemicals, such as sulfur-containing compounds, terpenoids, and polyphenols, which regulate the production of ROS and the levels of HDL and LDL in different ways to prevent atherosclerosis caused by *BRCA* mutations and thus prevent CVD, are shown in Figure 2.

## 6 Conclusion and outlook

PARPi and *BRCA* mutations play a significant role in the treatment of ovarian cancer. Clinicians are increasingly concerned about the side effects associated with PARPi and *BRCA* mutations. Phytochemicals, mostly derived from fruits and vegetables, have a high safety profile and are easily accessible, and therefore, patients have high compliance. In this study, we sorted out the principles of phytochemicals in antioxidants and maintenance of metabolic substance balance. We found that phytochemicals such as sulfur-containing compounds, polyphenols, and terpenoids can modulate the development of atherosclerosis, a key pathological change in the process of CVD caused by *BRCA* mutations, by mediating Keap1-Nrf2, free radicals, and LDL. In addition, phytochemicals can reduce the common clinical side effects of phytochemicals in reducing nausea and vomiting, relieving fatigue, and reducing hematotoxicity by modulating 5-HT, stimulating erythropoietin secretion, and antioxidant substances. We conclude that phytochemicals can inhibit the pathological changes caused by *BRCA* mutations and alleviate the side effects caused by PARPi by summarizing the relevant mechanisms. However, studies on phytochemicals that reduce the side effects of ovarian cancer treatment in animals are lacking, and natural phytochemicals are expected to gain wide usage in the clinical treatment of ovarian cancer.
